# Disorders of Puberty: Endocrinology of the Pre-Pubertal Testis

**DOI:** 10.3390/jcm9030780

**Published:** 2020-03-13

**Authors:** Sandro La Vignera, Rossella Cannarella, Rosita A. Condorelli, Aldo E. Calogero

**Affiliations:** Department of Clinical and Experimental Medicine, University of Catania, 95123 Catania, Italy; sandrolavignera@unict.it (S.L.V.); rossella.cannarella@phd.unict.it (R.C.); rosita.condorelli@unict.it (R.A.C.)

**Keywords:** Sertoli cells, Sertoli cell dysfunction, male infertility, inhibin B, AMH, IGF1, insulin

## Abstract

Male infertility is a widespread condition among western countries. Meta-regression data show that sperm concentration and total sperm count have halved in the last decades. The reasons of this decline are still unclear. The evaluation of testicular function in pre-pubertal children may be effective in the timely detection of Sertoli cell (SC) disfunction, which anticipates the diagnosis of male infertility. The aim of this Special Issue is to gather together in vitro evidence on SC physiology, causes of SC dysfunction, and to suggest a practical approach to be adopted in children.

The endocrinology of pre-pubertal testis represents a challenge for both endocrinologists and pediatricians because the testis has been believed to be dormant before the activation of the hypothalamic-pituitary-gonadal axis. However, various metabolic processes occur in the testis before the onset of puberty; these include proliferation of Sertoli cells (SC), secretion of anti-Müllerian hormone (AMH), and a slight increase in testicular volume. In particular, it is debated whether any of these parameters may be used as a useful diagnostic marker to identify early SC dysfunction, which probably anticipates the diagnosis of infertility in adulthood. This Special Issue focuses on the most recent advances in the endocrinology of the testis in pre-pubertal and transitional ages. The aim is to evaluate the physiology of pre-pubertal SCs and provide a proposal for the early detection of SC dysfunction. The structure of the Special Issue includes three reviews and seven original articles (including clinical and preclinical studies).

Pre-clinical studies mainly deal with the role of the growth hormone (GH)-insulin-like growth factor 1 (IGF1) axis on SCs. For these kinds of studies, SCs from pre-pubertal pigs were cultured. In contrast to adult SCs, pre-pubertal ones are immature, are able to proliferate, and can secret AMH and inhibin B hormones in the incubation medium. In the adult stage, SCs are mature, have lost the ability to proliferate, and therefore, these cells cannot be cultured. Pre-pubertal porcine SCs represent the in vitro system most similar to children’s SCs. Cannarella et al. report, for the first time, the role of the IGF1 receptor (IGF1R) in SCs, where they play a role similar to that already found in granulosa cells [1]. Other in vitro studies in this Special Issue [2,3] evaluate how incubation with follicle-stimulating hormone (FSH), GH, IGF1, or insulin impacts SC proliferation, AMH, and inhibin B secretion. Interestingly, these findings somehow question the role of FSH in SC proliferation in vitro, since no proliferative effect was found after 48 h of incubation. In contrast, both IGF1 and insulin enhanced SC proliferation. These results suggest that highly complex molecular mechanisms are involved in SC proliferation, and AMH and inhibin secretion in vivo. More than the effect of FSH alone, the increase in testicular volume and amount of circulating AMH and inhibin B in pre-pubertal children likely reflect a combination of multiple hormonal stimuli, among which IGF1 may play a relevant role. 

As far the clinical aspects, childhood cancer [4], pediatric varicocele [5], and risky lifestyles [6] (including substance abuse [7]) are addressed in the current Special Issue. Duca et al. […] evaluate the testicular function of childhood cancer survivors and address which cancer, therapy, and age of treatment has the worst reproductive outcomes in adulthood. This topic is of particular interest since the drugs used in pediatric oncology are very effective in terms of survival. Because childhood cancer survivors often will seek fertility later in life, it is wiser to use drugs with the lowest toxicity for the reproductive apparatus. The management of pediatric varicocele is somehow a debated issue. In the review by Cannarella et al., a general overview of pediatric varicocele is given, including a compelling flowchart reporting the management from an endocrinologic point of view [5]. Interestingly, a survey of the risky lifestyles for the reproductive and sexual function in male adolescents is provided by Perri et al. [6]. Worryingly, this study reveals a non-negligible percentage of smokers, drinkers, and cannabis consumers among male adolescents. In addition, many of them ignore sexual transmitted infections; proper information about risky health behaviors should be given. 

Therapeutic issues are also addressed in this Special Issue. These include the effectiveness of L-acetyl-carnitine for the treatment of asthenozoospermia in patients with type I diabetes mellitus [8] and the in vitro effects of thyroid hormones in sperm mitochondrial function, viability, and DNA integrity [9]. 

Finally, La Vignera et al. [10] discuss the diagnostic management that may be adopted to identify the early signs of isolated SC dysfunction. Therapeutic possibilities are also discussed. Overall, this study highlights the importance of carrying out well-designed prospective studies to validate the proposal made in the every-day clinical practice. Briefly, we suggest assessing pre-pubertal markers of testicular function (AMH and inhibin B) and testicular volume in patients with risk factors such as those detailed in Figure 1. Sperm analysis should not be requested earlier than 1.5 years of puberty onset [10]. As suggested in this article, measuring the response of AMH to stimulation with FSH, despite deserving a clinical validation, can represent a diagnostic test to promptly identify Sertolian dysfunction in the pre-pubertal age.

## Figures and Tables

**Figure 1:**
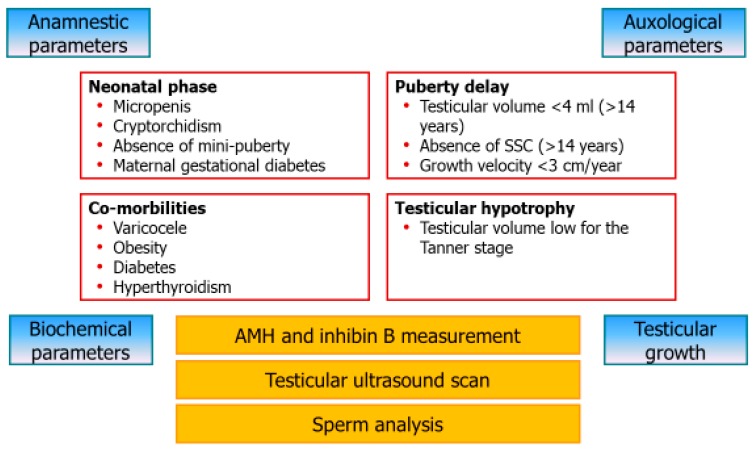
Diagnostic flow-chart for the early detection of Sertoli cell dysfunction. Children or transitional age adolescents showing anamnestic or physical signs at risk for Sertoli cell (SC) dysfunction should undergo to the assessment of biochemical parameters, testicular ultrasound and, whenever possible, sperm analysis.
